# Improvement of Theaflavins on Glucose and Lipid Metabolism in Diabetes Mellitus

**DOI:** 10.3390/foods13111763

**Published:** 2024-06-04

**Authors:** Shiyu Xu, Ying Chen, Yushun Gong

**Affiliations:** 1 National Research Center of Engineering and Technology for Utilization of Botanical Functional Ingredients, Changsha 410128, China; k9353785@kadai.jp; 2 Key Laboratory of Tea Science of Ministry of Education, Changsha 410128, China; 3 Co-Innovation Center of Education Ministry for Utilization of Botanical Functional Ingredients, Changsha 410128, China; 4 Key Laboratory for Evaluation and Utilization of Gene Resources of Horticultural Crops, Ministry of Agriculture and Rural Affairs of China, Hunan Agricultural University, Changsha 410128, China

**Keywords:** black tea, dietary polyphenols, insulin resistance, hyperglycemia, hypertriglyceridemia

## Abstract

In diabetes mellitus, disordered glucose and lipid metabolisms precipitate diverse complications, including nonalcoholic fatty liver disease, contributing to a rising global mortality rate. Theaflavins (TFs) can improve disorders of glycolipid metabolism in diabetic patients and reduce various types of damage, including glucotoxicity, lipotoxicity, and other associated secondary adverse effects. TFs exert effects to lower blood glucose and lipids levels, partly by regulating digestive enzyme activities, activation of OATP-MCT pathway and increasing secretion of incretins such as GIP. By the Ca^2+^-CaMKK ꞵ-AMPK and PI3K-AKT pathway, TFs promote glucose utilization and inhibit endogenous glucose production. Along with the regulation of energy metabolism by AMPK-SIRT1 pathway, TFs enhance fatty acids oxidation and reduce de novo lipogenesis. As such, the administration of TFs holds significant promise for both the prevention and amelioration of diabetes mellitus.

## 1. Introduction

Diabetes mellitus represents a persistent metabolic disorder marked by elevated blood glucose levels. According to the international diabetes federation (IDF), in 2021, there were approximately 537 million diabetics (aged 20–79), and the condition resulted in 6.7 million fatalities globally; by 2045, the number of diabetics will surge to about 783 million [1,2]. Type 2 diabetes mellitus (T2DM) is a heterogeneous disease caused by insulin resistance (IR) and insufficient insulin secretion, accounting for about 90–95% of all diabetes mellitus [1]. T2DM is associated with numerous complications, including hyperglycemia, hyperlipidemia, nonalcoholic fatty liver disease (NAFLD), diabetic nephropathy, and cardiovascular disease [3,4,5,6,7].

The pathogenesis of diabetes involves insulin deficiency (secretion and/or synthesis) and abnormal insulin response. Due to deficiencies in signaling molecules associated with mitochondrial metabolism and insulin signaling pathways, IR denotes impaired glucose utilization in liver and other major tissues [8]. Ectopic lipid deposition and elevated fatty acids (FA) levels mediate macrophage infiltration and promote the release of diacylglycerol (DAG), etc., thereby aggravating IR by activating protein kinase C (PKC)–insulin receptor–insulin receptor substrate (IRS) or inflammatory pathways. Abnormal pancreatic activity is associated with impaired proliferation of pancreatic ꞵ-cells, decreased insulin secretion, and increased glucagon secretion. During the initial phases of IR, insulin levels rise in a compensatory manner to sustain blood glucose levels. However, this is subsequently accompanied by impaired glucose-stimulated insulin secretion (GSIS) and apoptosis or dedifferentiation of pancreatic β-cells. These events contribute to abnormal pancreatic function and the onset of hyperglycemia [9,10,11]. In overnutrition, the elevated level of glucose and lipids predispose to glucotoxicity, lipotoxicity, and pancreatic dysfunction.

A multitude of pharmaceuticals are targeted at managing diabetes mellitus. For example, metformin shows antidiabetic effects by inhibiting intestinal glucose absorption and hepatic gluconeogenesis and reducing de novo lipogenesis (DNL), enhancing glucagon-like peptide 1 (GLP-1) secretion and promoting FA oxidation [12,13,14,15,16,17]. And novel drugs, DDP-4 inhibitors and SGLT-2 inhibitors, show the hypoglycemic effect by upregulating the endogenous GLP-1 level and inhibiting renal glucose reabsorption, respectively [18,19]. The aforementioned drugs carry risks of dose-related toxicity. Overuse or individual variability sometimes can heighten the likelihood of adverse effects such as weight gain, hypoglycemia, gastrointestinal disturbances, and lactic acidosis [20,21,22]. Research has indicated that drugs combination therapy demonstrates superior antidiabetic efficacy while minimizing side effects [23]. Dietary polyphenols, including compounds such as resveratrol and flavonoids, have been reported to possess therapeutic potential in blocking and ameliorating diabetes without notable side effects [24,25,26].

TFs are polyphenol oxidation products derived from black tea. In general, the concentration of TFs in black tea is about 0.3–3%. They are characterized by a benzodiazepine ketone structure and there are more than 28 derivatives of theaflavins (Table A1). Of these, Theaflavin (TF), Theaflavin-3-gallate (TF-3-G), Theaflavin-3′-gallate (TF-3′-G) and Theaflavin-3,3′-digallate (TF-D-G) are the most widely recognized. TFs possess various beneficial properties, including anti-inflammatory, antioxidative effects, and improvement of intestinal flora disorders, among others, which contribute to the prevention or improvement of tumors, hypertension, atherosclerosis and hepatic steatosis, etc. [27,28,29,30]. Besides, associated with the anti-obesity and anti-hypertriglyceridemia effects, TFs have shown a great potential to prevent and ameliorate diabetes mellitus, metabolic syndrome, and other complications [31,32,33,34]. This paper aims to elucidate the mechanism by which TFs balance glycolipid metabolism, thereby exerting preventive and ameliorative effects on diabetes.

## 2. Effects of TFs on Glucose Metabolism

### 2.1. Glucose Metabolism Disorders in Diabetes

Diabetes mellitus is characterized by hyperglycemia and glucose homeostasis dysregulation. Prediabetes is characterized by conditions such as impaired fasting glucose (IFG), IGT, or elevated Hemoglobin A1c (HbA1c) levels ranging from 5.7% to 6.4% (39 to 47 mmol/mol). In individuals diagnosed with diabetes, fasting plasma glucose (FPG) levels equal to or exceeding 126 mg/dL (7.0 mmol/L), or HbA1c levels equal to or exceeding 6.5% (48 mmol/mol), are typically observed [35].

Glucose homeostasis is mainly regulated by glucagon and insulin. In diabetics, the level of glucagon shows an absolute or relative increase. Initially, patients with T2DM may present with hyperinsulinemia [36]. Nonetheless, in later stages of T2DM and in type 1 diabetes mellitus (T1DM), insulin levels markedly decline due to pancreatic damage, resulting in degenerative alterations such as reductions in pancreatic cell numbers and sizes, central region vacancies, and exhaustion of pancreatic ꞵ-cells [37,38].

Glucose homeostasis dysregulation also involves abnormal glucose digestion and uptake, decreased glucose utilization, as well as increased endogenous glucose production (EGP) [39]. In individuals with T2DM, there is an elevation in the activity and abundance of sucrase and lactase enzymes. This increase enhances the absorption of monosaccharides in the intestine [40]. Sodium–glucose cotransporters (SGLTs) and glucose transporters (GLUTs) are the major glucose transporters. In T2DM patients, increased mRNA and protein expression of SGLT1, GLUT2, and GLUT5 promote the intestine uptake of glucose, galactose and fructose, respectively [41]. In the proximal renal tubules, elevated expression of SGLT2 facilitates the reabsorption of glucose in urine. Conversely, in liver and muscle tissues, there is a reduction in membrane translocation and protein expression of GLUT4, leading to inhibition of glucose uptake [42,43]. The body’s glucose level is regulated by glucose metabolism. On an empty stomach, liver glycogenolysis and gluconeogenesis produce glucose for energy. Postprandially, energy is provided by aerobic or anaerobic oxidation of glucose, and extra glucose is stored as glycogen in the liver and muscles. However, T2DM patients present decreased glycolysis and glycogen synthesis, along with increased gluconeogenesis, resulting in hyperglycemia and glucose metabolism disorders [44].

Hyperglycemia induces a range of secondary adverse effects, including dysbiosis of intestinal microbiota, intestinal damage, accumulation of advanced glycation end products (AGEs), and oxidative damage. Metagenome-wide association studies have revealed that T2DM is characterized by a decrease in bacteria responsible for producing short-chain fatty acids (SCFAs), such as *Lachnospiraceae*, alongside an increase in opportunistic pathogens such as *Verrucomicrobiaceae* [45,46]. Hyperglycemia has been indicated to change intracellular glucose metabolism and reprogramming by inducing reverse transport of glucose into intestinal epithelial cells via GLUT2, leading to intestinal barrier dysfunction and enteric infection. Moreover, the extent of intestinal permeability, known as leaky gut, correlates with the level of HbA1c in humans [47]. During T2DM, SREBP-1c upregulates the gene expression of GLUT2 [48]. Besides, hyperglycemia stimulates the generation of AGEs and enhances the binding of AGEs to the receptor of advanced glycation end products (RAGE). This process elevates the levels of reactive oxygen species (ROS) and inflammatory factors, increasing the risk of cardiovascular diseases and other related conditions [49,50,51]. Additionally, hyperglycemia upregulates the PKC hexosamine and polyol pathways to exacerbate oxidative stress, thereby inducing diabetic nephropathy and panvascular diseases [52].

### 2.2. TFs Ameliorate Diabetic Glucose Metabolism Disorders

Through the regulation of insulin and glucagon secretion, TFs effectively reduce blood glucose levels and enhance glucose homeostasis. Comparable to metformin, TFs decrease casual blood glucose, fasting blood glucose, and serum HbA1c levels, and ameliorate IGT in diabetic mice. Among TFs, TF3 exhibits the most potent hypoglycemic effect [53,54,55,56]. Clinical studies have reported that TFs significantly reduce the fasting and postprandial blood glucose level in prediabetics [54,57,58]. Further, a randomized trial has demonstrated that TFs exert a more pronounced hypoglycemic effect in younger individuals compared to older ones [59]. As regulators for blood glucose level, insulin and glucagon are respectively secreted by pancreatic ꞵ-cells and α-cells. TFs enhance the insulin activity significantly in vitro [60]. In obese rats, TFs decrease insulin levels, consequently improving hyperinsulinemia during the prediabetic period [32]. Meanwhile, in diabetic zebrafish, TFs improve abnormal pancreatic functions by promoting the regeneration of pancreatic ꞵ-cells and increasing their numbers. Lower concentrations of TF-D-G (5–10 μg/mL) have a better antidiabetic effect. In T2DM rats, TFs increase the number and functionality of the ꞵ-cells while inhibiting the proliferation of α-cells. Consequently, this leads to an elevation in insulin levels, a reduction in glucagon levels, and an improvement in IR (measured by HOMA-IR) [31,61,62,63]. The incretin promotes insulin secretion, inhibits glucagon secretion, and delays gastric emptying. In diabetic rats, TFs lower the blood glucose level by promoting the secretion of glucose-dependent insulinotropic polypeptide (GIP) and GLP-1 [56].

Dietary carbohydrates undergo enzymatic breakdown into monosaccharides, such as glucose, then enter intestinal epithelial cells. TFs exert their hypoglycemic effects by inhibiting carbohydrate digestion, thereby potentially reducing the absorption of glucose and other monosaccharides. In vivo and in vitro studies have revealed that TFs inhibit the activity of AGH, maltase, and α-amylase. This inhibitory effect varies in the following order: TF-D-G and TF-3-G > TF-3′-G > TF (related to the free hydroxyl group at the 3′position). The activity of saccharase and lactase are not affected by TFs [64,65,66,67,68].

### 2.3. Mechanisms Associated with the Improvement of Diabetic Glucose Metabolism Disorders by TFs

TFs regulate glucose intestinal absorption. Studies have shown that SGLT1 inhibition results in glucose and galactose malabsorption [69,70]. Through ^13^C_6_ glucose tracer assays conducted on Caco-2 cells, it has been observed that TFs activate the Ca^2+^/calmodulin-dependent protein β (Ca^2+^-CaMKK β) signaling cascade. This activation subsequently induces AMPK phosphorylation, leading to the downregulation of SGLT1 expression and inhibition of glucose uptake. Notably, the expression of SGLT2 and GLUT2 remains unchanged [71]. The urinary excretion of TFs accounts for approximately 94% of the intake due to considerably low bioavailability [72]. Via the organic anion transporting polypeptides (OATP) and monocarboxylic transporter (MCT) pathways, TFs enter intestinal epithelial cells. Subsequently, via ATP-binding cassette transporters (ABC transporters), TFs return to the gastrointestinal tract [73]. Through the OATP-MCT pathways, TFs effectively inhibit glucose uptake in intestinal epithelial cells [71].

TFs improve glucose metabolism disorders by enhancing glucose utilization and inhibiting EGP. The liver and skeletal muscles are the main organs associated with glucose metabolism. In IR HepG2 cells, TFs activate the IRS-PI3K-AKT pathway, leading to an increase in both total and membrane protein levels of GLUT4. This activation promotes glucose consumption by enhancing glucose uptake and improving mitochondrial biosynthesis [43]. In high-fat diet-induced obese mice, TFs were found to ameliorate hyperglycemia and IGT by increasing the membrane expression of GLUT4 and insulin receptors on skeletal muscle cells, as well as enhancing the activity of the β-subunit of insulin receptors [74,75]. In mature myotubular cells, TFs activate the Ca^2+^-CaMKK β-AMPK pathway, thereby enhancing glucose uptake and oxidative phosphorylation [76]. Glucose catabolism leads to the production of glycerol, among other molecules, for fat synthesis. In IR mice, TFs reduce membrane translocation of GLUT4 in adipocyte, inhibiting glucose-fat conversion [77]. Additionally, TFs regulate the activity of glucose metabolism-related enzymes. In diabetic zebrafish and rats, TFs significantly elevate the key enzyme activity of glycolysis (hexokinase and pyruvate kinase), glycogen synthase, and glucose-6-phosphate dehydrogenase (the rate-limiting enzyme of the pentose phosphate pathway), and decrease the enzyme activity of gluconeogenesis (glucose-6-phosphatase, fructose-1,6-bisphosphatase, and PEPCK), glycogen phosphorylase, and lactate dehydrogenase [31,37,61]. In HL1c hepatocytes, TFs induce the phosphorylation of FOXO1a, thus inhibiting the gene and protein expression of PEPCK. This effect occurs independently of insulin or insulin-like growth factor-1 (IGF-1), with an IC50 of 20 μg/mL^−1^ [78].

TFs ameliorate intestinal damage, while improving microbiota dysbiosis and other secondary adverse effects caused by glucotoxicity. In Caco-2 cells, TFs upregulate the mRNA and protein expression of tight junction proteins, such as Cingulin, Occludin, Claudin-1, and ZO-1, to enhance intestinal barriers [79]. In drosophila and mice, by interacting with intestinal flora, TFs were found to alleviate metabolic toxins, including ammonia and methylglyoxal (MGO), thereby ameliorating intestinal leakage and microbiota dysbiosis [27,80]. Notably, TF-D-G has been shown to modulate the composition of gut flora following intraperitoneal injection. As revealed by 16S rRNA sequencing data, TF-D-G improves dysbiosis of intestinal microbiota by increasing the abundance of *Prevotellaceae*, *Ruminococcaceae* and other beneficial bacteria, while concurrently decreasing the abundance of *Parvibacter* and other opportunistic pathogens [81]. In diabetic mice, TFs were found to reduce the levels of AGEs precursor MGO and malondialdehyde (MDA). Further, they inhibit the accumulation of AGEs, ROS, and inflammatory factors, thereby ameliorating diabetes mellitus and its complications, such as diabetic nephropathy. Moreover, TFs have been shown to be more effective than metformin at the same oral dose (150 mg/kg) [55,82,83]. In T2DM patients, TFs were found to upregulate the mRNA and protein expression of sirtuin1 (SIRT1), thereby enhancing insulin sensitivity, inhibiting hyperglycemia, and reducing oxidative stress [84].

### 2.4. Summary

**Figure 1:**
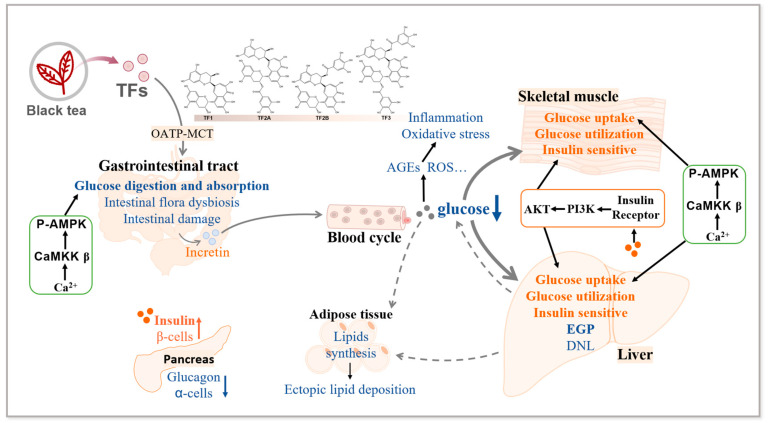
The effect of TFs on glucose metabolism disorders. TFs from black tea enter intestinal epithelial cells through the OATP-MCT pathway, then induce an increase in Ca^2+^ concentration. The activation of Ca^2+^-CaMKK ꞵ-AMPK pathway and OATP-MCT pathway inhibit glucose digestion and absorption. Besides, TFs improve intestinal flora dysbiosis and damage, as well as promoting incretin secretion. In the pancreas, TFs promote β-cells proliferation and insulin secretion and inhibit α-cells proliferation and glucagon secretion, thus inhibiting circulating blood glucose levels. By increasing the insulin level and insulin receptors activity, TFs enhance insulin sensitivity. Through the Ca^2+^-CaMKK ꞵ-AMPK pathway, TFs promote glucose uptake and glucose utilization in liver and skeletal muscle. Along with inhibited EGP and DNL, TFs reduce lipids transportation from liver to adipose tissue. In adipose tissue, TFs inhibit the conversion of glucose to fat and thus decrease ectopic lipid deposition. TFs reduce circulating blood glucose levels, inhibit the production of secondary adverse products such as AGEs and ROS, and inhibit inflammation and oxidative stress, thereby improving glucose metabolism disorders. OATP: organic anion-transporting polypeptides; MCT: monocarboxylic transporter; EGP: endogenous glucose production; DNL: de novo lipogenesis. A solid line and the orange color indicate enhancement; a dashed line and the blue color indicate suppression.

Diabetes mellitus is characterized by disturbances in glucose metabolism, manifested by increased EGP and decreased glucose utilization. TFs effectively lower short-term blood glucose levels (fasting and postprandial), as well as long-term levels (HbA1c), and improve IGT. Additionally, they regulate insulin and glucagon secretion, both directly and indirectly. Moreover, TFs ameliorate intestinal damage, intestinal flora dysbiosis, and secondary adverse effects caused by glucotoxicity. TFs also improve glucose metabolism disorders by inhibiting glucose digestion and absorption, regulating glucose uptake, inhibiting EGP (gluconeogenesis and glycogenolysis), and facilitating glucose utilization (glycolysis and glycogen synthesis) (Figure 1).

## 3. Effects of TFs on Lipid Metabolism

### 3.1. Lipid Metabolism Disorders in Diabetes

Diabetes is usually associated with hyperlipidemia and local obesity. In diabetics, the serum total cholesterol (TC), triglyceride (TG), and low-density lipoprotein (LDL) levels increase, and the high-density lipoprotein (HDL) level decreases. This metabolic imbalance leads to a redistribution of body fat from subcutaneous deposits to visceral fat, resulting in central obesity, enlarged liver and adipose tissue, and increased lipid droplets. During the early stages of T2DM, patients often exhibit an obese body shape due to excessive fat accumulation induced by IR. Conversely, individuals with T1DM and those in the later stages of T2DM may present with an emaciated body shape due to a shift in energy consumption from glucose to lipids and proteins, which is caused by insulin deficiency. 

Adipose tissues serve as a specialized depot for energy storage in the form of fat and facilitate energy mobilization through fat mobilization. Additionally, they function as an endocrine organ, secreting adipokines. Diabetes mellitus is characterized by disordered lipid metabolism, including abnormal lipid absorption, heightened lipid synthesis and fat mobilization, reduced lipid consumption, as well as ectopic lipid deposition and disruptions in energy metabolism. Patients with T2DM have been reported to exhibit elevated apolipoprotein B (ApoB) and non-esterified fatty acids (NEFAs) levels, as well as increased production and delayed catabolism of lipoproteins, for example, chylomicron, leading to postprandial hyperlipidemia [85,86,87]. The liver is a vital organ for FA metabolism and lipid synthesis. In diabetic livers, fat anabolism is stronger than fat oxidation due to IR. At the same time, the enhanced cholesterol synthesis promotes lipids accumulation, which exacerbates metabolic syndrome and atherosclerosis [88,89]. When the lipid content in hepatocytes exceeds 5%, hepatic steatosis occurs [90]. Studies have reported that lowering lipid levels can restore insulin sensitivity in the liver [91]. In the pancreas, lipid accumulation can lead to the development of chronic pancreatitis, glucose metabolism disorders, and impairment of insulin secretion [92]. However, reducing lipid levels has been shown to ameliorate the pancreatic damage associated with these conditions [93]. Besides, in obesity and T2DM patients, high-fat-induced abnormal mitochondrial function results in disturbed energy metabolism [94,95].

Diabetic lipid metabolism disorders also involve intestinal flora dysbiosis and impaired intestinal barriers. In the intestines of individuals with T2DM, there is a reduction in the abundance of bacteria that produce SCFAs, while the level of bacteria that produce LPS increases. This dysbiosis is accompanied by impaired intestinal barriers. Elevated levels of circulating LPS induce endotoxemia and hyperinflammatory responses [96,97]. Additionally, dysbiosis of the intestinal flora also results in a reduction in the conversion of primary bile acids to secondary bile acids. Disturbed bile acid metabolism raises the risk for intestinal diseases. 

Simultaneously, diabetic lipid metabolism disorders are exacerbated by lipotoxicity and lipid peroxidation. In the pancreas, liver, skeletal muscle, and other tissues, ectopic lipid deposition fosters the buildup of ceramide and amyloid, thereby triggering lipotoxicity. Lipotoxicity triggers chronic inflammation, oxidative stress, endoplasmic reticulum (ER) stress, autophagy, and even apoptosis, which then results in diabetes mellitus, NAFLD, and renal disease, as well as other conditions [98,99]. Pancreatic cells are particularly vulnerable to changes in fat levels and lipotoxicity [100]. Elevated levels of saturated fatty acids (SFAs) promote increased production of ceramide, cytochrome C, and DNA fragmentation, subsequently leading to apoptosis, inhibition of proliferation, and impairment of insulin secretion in the pancreas [100,101]. Notably, unsaturated fatty acids (UFAs) exhibit the opposite effect. Elevated levels of FA and abnormal adipokines contribute to mitochondrial dysfunction and inflammation, resulting in attenuated FA ꞵ-oxidation, increased ROS production, and oxidative stress [102]. Diabetes promotes the accumulation of lipid peroxides such as 4-hydroxy-trans-2-nonenal (4-HNE), leading to organ damage. Ferroptosis, which is characterized by iron-dependent accumulation of lipid peroxides, is a form of cell death. In diabetes, ferroptosis exacerbates ER stress, impaired insulin biosynthesis, and β-cell death by inhibiting the expression of oxidoreductases such as glutathione peroxidase (GPX4), and the interplay of iron–sulfur (Fe-S) clusters [103].

### 3.2. TFs Ameliorate Diabetic Lipid Metabolism Disorders

TFs have been shown to improve hyperlipidemia and regulate body composition. Several studies have demonstrated that TFs exhibit a superior lipid-lowering effect compared to other tea polyphenols, such as EGCG. Further, TF-D-G has been identified as having the most potent hypolipidemic effect among the four monomers, with the least toxicity [104]. According to JIN D et al., in obese rats, TFs reduce the adiposity index by 24.5%, and significantly decrease serum levels of TC, TG, and LDL by 26.5%, 50.8%, and 71.7%, respectively. This demonstrates visible anti-obesity and lipid-lowering effects [32]. Clinical trials have shown that oral administration of TFs to hypercholesterolemia patients reduces the serum levels of TC, TG and LDL, and improves the body composition by proportionally decreasing the total and subcutaneous fat and increasing skeletal muscle [57,105,106,107,108].

The hypolipidemic effect of TFs is linked to the type of diabetes. In obese mice and patients with T2DM, TFs lead to reductions in body weight, waist circumference, and fat content in tissues such as the mesentery, epididymis, and liver [54,81]. Meanwhile, in slim T2DM mice and patients, TFs suppress body-weight loss [31,55]. Several studies have reported that the anti-obesity effect of TFs is partly attributed to appetite suppression and reduced food intake [32,53], while others have indicated that TFs do not affect food intake [54]. Further, different treatment concentrations of TFs have varying effects on food intake and feed efficiency: lower concentrations have been shown to promote food intake, whereas higher concentrations have an inhibitory effect [81]. This suggests that the effect of TFs on food intake may not be solely responsible for their anti-obesity effect. Additionally, TFs exert their anti-obesity effect and improve diabetes mellitus by increasing fecal TG excretion and alleviating diabetic symptoms such as polydipsia and polyuria [31,55,109].

### 3.3. Mechanisms Associated with the Improvement of Diabetic Lipid Metabolism Disorders by TFs

TFs inhibit lipid digestion and absorption. Dietary lipids, such as TG, phospholipids and cholesterol esters are hydrolyzed by lipases to produce NEFAs, 2-monoacylglycerols, lysophospholipids, and non-esterified cholesterol, which are then absorbed by the intestine. Pancreatic lipase (PL) is a lipase enzyme that is synthesized and secreted by the pancreas, being responsible for 50–70% of dietary lipid digestion [110]. In vivo and in vitro studies have revealed that TFs inhibit PL activity in a non-substrate-competitive manner. TF-D-G shows a similar inhibitory effect to orlistat, a clinical PL inhibitor [111,112,113,114]. On the other hand, TFs also improve pancreatic functions and thus upregulate the gene expression of carboxyl ester lipase (CEL), pancreatic triglyceride lipase (PNLIP), chymotrypsinogen B1 (CTRB1), and chymotrypsin-like elastase 3B (CELA3B) [33]. In the intestines of individuals with T2DM, TFs promote the secretion of intestinal hormones, such as GLP-1. This leads to a reduction in postprandial TG and NEFAs levels, as well as a decrease in ApoB levels. Further, TFs inhibit chylomicron production and directly reduce the expression of genes related to lipoprotein metabolism [115,116,117]. In Caco-2 cells, TFs competitively bind to the apical sodium-dependent bile acid transporter (ASBT) to inhibit the taurocholic acid uptake and lower the plasma cholesterol level [118]. TFs have been found to be more effective than EGCG in directly inhibiting the formation of dietary mixed micelles, which consist of mixed oleic acid, bile acids, cholesterol, and others [119]. The inhibitory effect leads to a reduction in cholesterol absorption, and this effect is dose dependent. Moreover, TFs inhibit postprandial hypertriglyceridemia by restoring the lymphatic uptake of TG [113].

TFs ameliorate lipid metabolism disorders by balancing energy metabolism. TFs have been observed to not affect the locomotor activity of mice; however, they increase oxygen consumption (VO2) and energy expenditure (EE). This suggests that TFs exhibit an anti-obesity effect by promoting lipid oxidation. Specifically, TFs upregulate the mRNA levels of uncoupling protein-1 (UCP-1) and UCP-3 in brown adipose tissue and gastrocnemius muscle, further enhancing lipid oxidation [120].

TFs also improve lipid metabolism disorder by inhibiting lipid synthesis and catabolism, as well as promoting lipid oxidation. TFs induce the phosphorylation of AMPK by LKB1 and ROS pathways, subsequently enhancing the expression of SIRT1. This process inhibits the nuclear translocation of SREBP-1, thereby modulating lipid metabolism. Consequently, TFs decrease the mRNA and protein expression of fatty acid synthase (FAS), acetyl-CoA carboxylase (ACC) and HMG-CoA reductase (HMGCR), while increasing the expression of carnitine palmitoyl transferase 1 (CPT1), thereby inhibiting FA and cholesterol synthesis, decreasing lipid accumulation, as well as promoting FA oxidation [53,121,122,123,124,125]. Additionally, by decreasing hepatic lipase (HL) activity, TFs were found to inhibit fat mobilization in obese rats [32]. Specificity protein 1 (Sp1) is a transcription factor. Blocking of the Sp1 binding site inhibits cell proliferation and FAS expression [126]. By downregulating the EGFR-PI3K-AKT-Sp1 pathway, TFs inhibit lipid synthesis [122].

By affecting other downstream targets of SIRT1, TFs balance the energy metabolism and lipid metabolism. Peroxisome proliferator-activated receptor-γ coactivator-1 (PGC-1) plays a key role in mitochondrial biosynthesis and energy metabolism, namely, PGC-1α, PGC-1β and PGC-1-related coactivator (PRC). In T2DM mice and HepG2 cells, TFs were found to significantly upregulate the mRNA levels of PGC-1α and PRC and downregulate the mRNA level of PGC-1β. This was found to promote mitochondrial biosynthesis and FA oxidation by increasing mitochondrial abundance, mt DNA copy number, and mitochondrial respiratory chain complex V (F_1_F_0_-ATPase) activity [33,43].

TFs regulate cholesterol metabolism in various ways. The liver plays a pivotal role in cholesterol metabolism, as it is interconnected with the intestines through bile acids and bioactive substances. In the ileum, TFs inhibit the mRNA and protein expression of FXR and FGF15, as well as suppressing the expression of genes related to cholesterol metabolism such as CYP7B1, CYP27A1, and FXR. Moreover, TFs redirect the classical bile acid biosynthesis pathway towards an alternative pathway, resulting in a significant improvement in cholesterol deposition [127]. Experiments on obese NAFLD mice have revealed that TFs upregulate the lipid metabolism related genes expression, such as UFA biosynthesis (Fads1, Tecr, Scd1, and Elovl1), arachidonic and linoleic acid metabolism (CYP4F14, CYP1A2, and CYP2C70), and steroid biosynthesis (Fdft1, Tm7sf2, Ebp, and Dhcr7), while downregulating the gene expression of the PPAR signaling pathway (Fabp4, Plin4, Lpl, and Acadm). In parallel, by activating the Fads1-PPARδ-Fabp4 pathway, TFs regulate lipid metabolism and reduce foam cell formation induced by VLDL [81,128].

TFs also ameliorate lipid metabolism disorders by regulating hepatokines and adipokine secretion. By lowering ALT and AST levels, TFs improve liver functional damage [53]. Plasma kallikrein (PK), a trypsin-like serine protease specifically synthesized and secreted by the liver, is crucial for the differentiation of 3T3-L1 cells into adipocytes. PK inhibition contributes to anti-inflammatory effects and reductions in lipid levels [129]. TFs directly inhibit PK activity, thereby alleviating hepatic lipid deposition [104]. Adipokines are proteins secreted by adipocytes, including lipocalin, leptin, IL-6, and TNF [130]. Among these, lipocalin and leptin are insulin sensitizers, with lipocalin reducing hepatic gluconeogenesis and increasing FA oxidation, which is negatively correlated with adiposity [131,132]. Conversely, leptin increases the incidence of metabolic syndrome and diabetes, which are positively correlated with adiposity. In rats with fructose-induced hyperlipidemia and hyperleptinemia, TFs increase circulating lipocalin levels and decrease leptin levels, thereby lowering lipid levels and improving IR. Moreover, long-term intake of TFs in humans with obesity, overweight, and T2DM exhibits similar modulatory effects on adipokines [123,133,134].

TFs inhibit the generation and accumulation of endotoxin, lipotoxicity, and lipid peroxides. Elevated levels of endotoxins stimulate the accumulation of cytokines such as TNF-α and inflammatory mediators like NO, thereby inducing chronic inflammation. By blocking NF-κB nuclear translocation and JNK phosphorylation, TFs reduce endotoxin and inflammatory factors levels while improving intestinal leakage and intestinal epithelial damage in colitis mice [27,135]. Both lipotoxicity and lipid peroxidation are related to abnormal lipid oxidation. In diabetic rats, TFs increase the levels of enzymatic and non-enzymatic antioxidants, including superoxide dismutase (SOD), catalase (CAT), GPX4, glutathione S-transferase (GST), and reduced glutathione (GSH). In parallel, TFs reduce the levels of hydrogen peroxide and ROS, thereby ameliorating lipotoxicity, along with restoring mitochondrial function and pancreatic function [31,136]. MicroRNAs (miRNAs) are small non-coding RNAs that regulate various cellular activities in post-transcriptional regulation [137]. By inhibiting the expression of miR-128-3p to facilitate the activation of SIRT1 or increasing the expression of miR-24 to facilitate the activation of the Nrf2-NQO1/HO-1 pathway, TFs suppress oxidative stress and apoptosis triggered by lipotoxicity [138,139,140]. Moreover, by diminishing the formation of β-folded structures, TFs impede the generation and buildup of amyloid β, thereby reducing lipotoxicity [141,142,143]. TFs inhibit the production and accumulation of lipid peroxidation products, such as thiobarbituric acid-reactive substances (TBARS), acrolein and 4-HNE, thereby ameliorating the functional and structural cell damage caused by lipid peroxidation [144]. Further, by activating the Nrf2-GPX4 signaling pathway, TFs upregulate the expression of GPX4, HO-1 and FTH1, while inhibiting the accumulation of mitochondrial ROS and Fe^2+^, thereby improving lipid peroxidation-induced ferroptosis [145].

### 3.4. Summary

**Figure 2:**
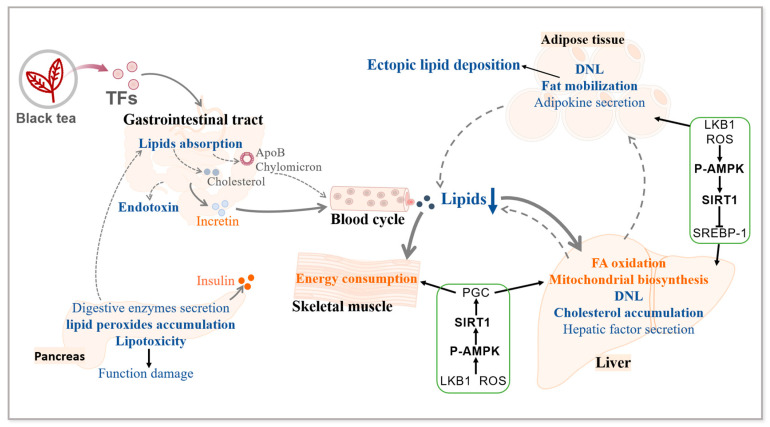
The effect of TFs on lipids metabolism disorders. TFs extracted from black tea inhibit lipids digestion and absorption, decrease endotoxin secretion, and increase incretin levels. By inhibiting lipid peroxides accumulation and lowering lipotoxicity, TFs improve pancreatic functions and thus increase insulin secretion. Through LKB1 and ROS pathways, TFs induce the activation of AMPK-SIRT1-PGC pathway, thereby lowering lipids levels by improving mitochondrial biosynthesis and enhancing energy consumption in the muscle and liver. The activation of SIRT1 inhibits SREBP-1, promotes FA oxidation, and reduces DNL, cholesterol accumulation, and fat mobilization in liver and adipose tissue, thereby decreasing circulating lipids levels and ectopic lipid deposition. DNL: de novo lipogenesis. A solid line and the orange color indicate enhancement; a dashed line and the blue color indicate suppression.

Diabetes mellitus is characterized by lipid metabolism disorders, along with decreased lipid consumption, increased lipid synthesis, and ectopic lipid deposition. TFs exert an anti-obesity effect by ameliorating hyperlipidemia (lowering TC, TG, and LDL levels) and modulating body composition. Meanwhile, TFs modulate hepatokines and adipokines (adiponectin and leptin) levels, inhibit endotoxins, and reduce lipotoxicity and lipid peroxidations. TFs have demonstrated the ability to ameliorate lipid metabolism dysfunction through various mechanisms, including modulation of lipid digestion and absorption, regulation of energy metabolism, inhibition of lipid synthesis, and promotion of lipid oxidation (Figure 2).

## 4. Conclusions

**Figure 3:**
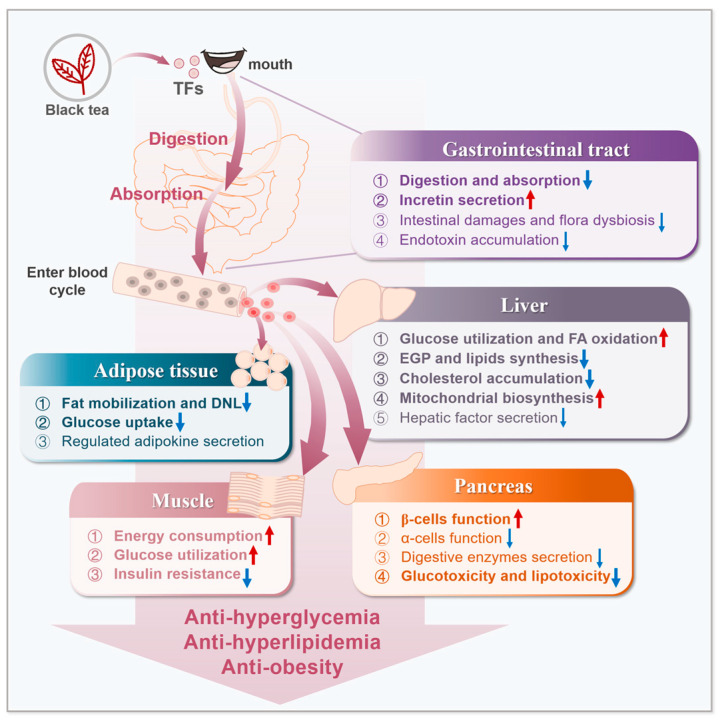
The effects of TFs on glucose and lipid metabolism disorders. TFs extracted from black tea inhibit digestion and absorption and increase incretin levels. Through the blood cycle, TFs increase mitochondrial biosynthesis and thus enhance energy consumption. In liver, TFs promote glucose utilization and FA oxidation. By decreasing EGP, lipids synthesis, cholesterol accumulation, fat mobilization, etc., TFs show the hypoglycemic and hypolipidemic effects. What is more, TFs improve pancreatic functions by lowering glucotoxicity and lipotoxicity, thereby increasing the insulin levels and inhibiting insulin resistance. TFs exert anti-hyperglycemia, anti-hyperlipidemia, and anti-obesity properties, and have potential as a dietary supplement for improving disorders of glucose and lipid metabolism. The blue arrowhead indicates inhibition by TFs, and red one indicates enhancement by TFs.

TFs are vital, quality ingredients of black tea. By augmenting glucose utilization and enhancing lipids oxidation, as well as restraining hepatic EGP and lipids synthesis, TFs alleviate diabetic glycolipid metabolism abnormalities (Figure 3). TFs heighten insulin sensitivity by stimulating the insulin receptor-IRS-PI3K-AKT pathway. Via upregulation of Ca^2+^ and ROS levels or stimulating LKB1, TFs activate the AMPK-SIRT1 pathway, thereby recovering balance of energy metabolism and glycolipid metabolism. Besides, TFs contribute to decreases in the level of AGEs, endotoxins, and lipid peroxides, thus diminishing cell damage, improving pancreatic function, and suppressing oxidative stress and inflammation. 

## 5. Future Directions

TFs can modulate glucose and lipid metabolisms through shared substrates. Dihydroxyacetone phosphate (DHAP) functions as a common substrate in glycolipid metabolism. By the DNL pathway or the gluconeogenesis pathway, it can synthesis TG or glucose-6-phosphate, respectively. Glycerol-3-phosphate dehydrogenase (GPDH) is a key enzyme of DNL, which catalyzes the generation of glycerol-3-phosphate from DHAP. Studies have shown that tea polyphenols such as EGCG reduce GPDH activity and inhibit lipid synthesis in a dose-dependent manner [146]. TFs and EGCG exhibit similar efficacy while TFs show higher safety [147]. It is hypothesized that TFs may inhibit gluconeogenesis and DNL while reducing ectopic lipid deposition by decreasing the GPDH activity. 

TFs may prevent and ameliorate T2DM by promoting the synthesis or secretion of intestinal hormones. Intestinal hormones, such as GLP-1, GIP, and cholecystokinin (CCK), have been demonstrated to influence the progression of T2DM by modulating glycolipid metabolism [148,149,150,151,152,153]. Gut hormone coactivators, exemplified by GLP-1/GIP receptor co-agonists, are utilized in the clinical management of T2DM due to their efficacy in promoting weight loss and lowering lipid and blood glucose levels while exhibiting minimal side effects [154]. Human bitter taste-sensing type 2 receptors (hTAS2Rs), present in the oral cavity and the pancreas, play a role in stimulating the secretion of intestinal hormones [155,156,157]. It has been reported that in HEK293T cells, TFs enhance the expression of hTAS2R39 and hTAS2R14 in a dose-dependent manner [158]. Thus, the investigation of whether TFs enhance the secretion of intestinal hormones by activating the hTAS2Rs cascade pathway warrants further research.

**Figure 4:**
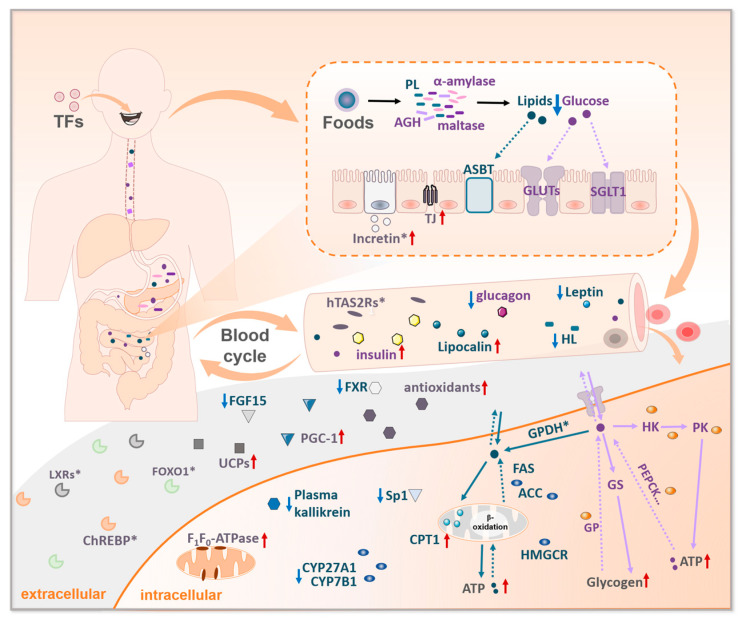
TFs affect glucose-lipid metabolism-related proteins and hormones. PL: pancreatic lipase; AGH: α-glucosidase; TJ: tight junction protein; ASBT: apical sodium-dependent bile acid transporter; HL: hepatic lipase; PGC-1: proliferator-activated receptor-γ coactivator-1; UPC: uncoupling protein; Sp1: specificity protein 1; CPT1: carnitine palmitoyl transferase 1; FAS: fatty acid synthase; ACC: acetyl-CoA carboxylase; HMGCR: HMG-CoA reductase; HK: hexokinase; PK: pyruvate kinase. GP: glycogen phosphorylase; GS: glycogen synthase; “*” means more research is still needed. A solid line and the red arrowhead indicate enhancement; a dashed line and the blue arrowhead indicate suppression.

A number of studies have shown that TFs regulate nuclear transcription factors related to glucose–lipid metabolisms, including liver X receptors (LXRs), FOXO1 and carbohydrate responsive element binding proteins (ChREBP). Interestingly, all the above factors are associated with liver. LXR activators hold promise as potential therapeutics for T2DM for the effects of inhibiting gluconeogenesis and enhancing adipogenesis [159,160,161,162]. Computerized molecular modeling has shown that TFs are potential LXR-ꞵ activators [163]. FOXO1 enhances gluconeogenesis and liposynthesis and increases the level of apolipoproteins (apo C-III. and microsomal triglyceride transporter proteins), thereby facilitating VLDL accumulation in vivo [164]. Moreover, FOXO1 is a negative regulator for hepatic insulin signaling. TFs have been shown to inhibit gluconeogenesis through the PI3K-Akt-FOXO1 pathway [78,165]. As such, the efficacy of TFs in regulating glucose-lipid metabolism by inhibiting the FOXO1 pathway deserves further investigation. ChREBP is a central regulator of glycolysis and DNL, and modulates glucotoxicity and lipotoxicity [166]. ChREBP is activated by a key enzyme of glucose metabolism (glucose-6-phosphatase and fructose-2,6-bisphosphatase) and glycolipid toxicity-associated products (AEGs, etc.). TFs affect the expression of the aforementioned ChREBP-related activators. Thus, ChREBP emerges as a potential factor through which TFs regulate glucose-lipid metabolism disorders [167,168]. TFs affect a lots of proteins and hormones to balance diabetic glycolipid metabolisms (Figure 4).

Studies have reported that the Papp values of TFs range from 0.44 × 10^−7^ to 3.64 × 10^−7^ cm/s in the absorptive transport and the efflux ratio is over 1.24 [169]. In humans, 2 h after 700 mg intake of TFs, the maximum concentration in the blood plasma is 1.0 μg/L [170]. The low bioavailability of TFs is partly due to the structural instability, efflux transporters, and cell metabolism, etc. The model of the Caco-2 monolayer has shown that the cytotoxicity of TFs is in the order of TF-3′-G > TF-D-G > TF-3-G > TF, and the stability (pH = 6) is in the order of TF > TF-D-G > TF-3-G/TF-3′-G, which is affected by the galloyl moiety [169]. High concentrations of TFs damage cell viability. In either the upper or lower gastrointestinal tract, theaflavins are not absorbed. Mainly in the colon, the galloyl moiety of TFs is broken down by microbiota to gallic acid, which is then converted to 3-O- and 4-O-methyl gallic acids, pyrogallol-1-sulfate and pyrogallol-2-sulfate, etc. [72]. These colonic catabolites have a variety of potential biological activities in vitro and in vitro, which may contribute to various efficacies of TFs. Gut microbiota plays a crucial role in the biotransformation and activities of TFs, and further research is needed on this topic [171]. Additionally, theaflavins have been found to be bioavailable in liver and prostate in conjugated and free forms, and may be capable of preventing prostate cancer [172]. Recent studies have reported that nanocomplexes are potentially able to enhance the intestinal absorption of TFs [173]. According to mice or rat experiments, 150 mg/kg is the optimal dosage, and further research is still needed to determine the optimal dosage, formulation, or delivery methods in humans. TFs exert biological active in a specific concentration range and purity. Therefore, the separation and purification of TFs is vital. 

## Data Availability

The original contributions presented in the study are included in the article, further inquiries can be directed to the corresponding authors.

## Appendix A

**Table A1:** Theaflavin derivatives, precursors, and structural formulas [174,175].

	Theaflavin Derivatives	Precursors	Structural Formulas
1	Theaflavin (TF)	EC + EGC	(1) R1 = R4 = OH, R2 = R3 = H (2) R1 = R3 = H, R2 = R4 = OH (3) R1 = R3 = OH, R2 = R4 = H (4) R1 = OH, R2 = R3 = H, R4 = gallate (5) R1 = R3 = H, R2 = OH, R4 = gallate (6) R1 = gallate, R2 = R3 = H, R4 = OH (7) R1 = gallate, R2 = R4 = H, R3 = OH (8) R1 = R4 = gallate, R2 = R3 = H
2	Isotheaflavin B (TF1b)	EC + (+)-GC	
3	Neotheaflavin C (TF1c)	(+)-C + EGC	
4	Theaflavin-3-gallate (TF-3-G)	EC + EGCG	
5	Neotheaflavin-3-gallate	(+)-C + EGCG	
6	Theaflavin-3′-gallate (TF-3′-G)	ECG + EGC	
7	Isotheaflavin-3′-gallate	ECG + (+)-GC	
8	Theaflavin-3,3′-gallate (TF-D-G)	ECG + EGCG	
9	Theaflavic acid	(+)-EC + GA	(9) R1 = OH, R2 = H (10) R1 = H, R2 = OH (11) R1 = H, R2 = gallate
10	Epitheaflavic acid	(-)-EC + GA	
11	Epitheaflavic acid-3-gallate	(-)-ECG + GA	
12	Theaflagallin	(±)-GC + GA (Pyrogallol)	(12) R1 = OH, R2 = H, R3 = OH (13) R1 = H, R2 = OH, R3 = OH (14) R1 = H, R2 = gallate, R3 = OH (15) R1 = OH, R2 = H, R3 = H (16) R1 = gallate, R2 = R3 = H
13	Epitheaflgallin		
14	Epitheaflagallin-3- gallate	EGCG + GA (Pyrogallol)	
15	Undecided	EGC + Catechol	
16	Undecided	EGCG + Catechol	
17	Purpurogallin	Pyrogallol	(17) R1 = H, R2 = OH (18) R1 = COOH, R2 = OH (19) R1 = COOH, R2 = H
18	Purpurogallin carboxylic acid	GA + GA (Pyrogallol)	
19	Undecided	GA + Catechol	
20	Theaflavate A	ECG	(20) R1 = gallate, R2 = H (21) R1 = OH, R2 = H (22) R1 = H, R2 = OH
21	Theaflavate B	ECG + EC	
22	Neotheaflavate B	C + EGCG	
23	Theadibenzotropolone A	Theaflavin-3-gallate + EC	(23) R1 = H, R2 = OH, R3 = H, R4 = OH (24) R1 = OH, R2 = H, R3 = H, R4 = OH (25) R1 = H, R2 = OH, R3 = OH, R4 = H
24	Theadibenzotropolone B	Theaflavin-3-gallate + C	
25	Theadibenzotropolone C	Neotheaflavin-3-gallate + EC	
26	Theatribenzotropolone A	Theaflavin-3,3′-gallate + 2EC	
27	Bistheaflavin A	Theaflavin + Theaflavin	
28	Bistheaflavin B	Theaflavin + Theaflavin	

Note: EC: Epicatechin; ECG: Epicatechin gallate; EGC: epigallocatechin; EGCG: epigallocatechin gallate; GA: gallic acid; C: catechin; CG: catechin gallate; GC: gallocatechin.
